# Large-scale analysis of chromosomal aberrations in cancer karyotypes reveals two distinct paths to aneuploidy

**DOI:** 10.1186/gb-2011-12-6-r61

**Published:** 2011-06-29

**Authors:** Michal Ozery-Flato, Chaim Linhart, Luba Trakhtenbrot, Shai Izraeli, Ron Shamir

**Affiliations:** 1 The Blavatnik School of Computer Science, Tel Aviv University, Tel Aviv, 69978, Israel; 2 Machine Learning and Data Mining Group, IBM Haifa Research Lab, Mount Carmel, Haifa, 31905, Israel; 3 Chaim Sheba Cancer Research Center, Sheba Medical Center, Tel Hashomer, Ramat Gan, 52620, Israel; 4 Institute of Hematology, Sheba Medical Center, Tel Hashomer, Ramat Gan, 52620, Israel; 5 Department of Pediatric Hemato-Oncology, Edmond and Lily Safra Children's Hospital, Sheba Medical Center, Tel Hashomer, Ramat Gan, 52620, Israel; 6 Sackler School of Medicine, Tel Aviv University, Tel Aviv, 69978, Israel

## Abstract

**Background:**

Chromosomal aneuploidy, that is to say the gain or loss of chromosomes, is the most common abnormality in cancer. While certain aberrations, most commonly translocations, are known to be strongly associated with specific cancers and contribute to their formation, most aberrations appear to be non-specific and arbitrary, and do not have a clear effect. The understanding of chromosomal aneuploidy and its role in tumorigenesis is a fundamental open problem in cancer biology.

**Results:**

We report on a systematic study of the characteristics of chromosomal aberrations in cancers, using over 15,000 karyotypes and 62 cancer classes in the Mitelman Database. Remarkably, we discovered a very high co-occurrence rate of chromosome gains with other chromosome gains, and of losses with losses. Gains and losses rarely show significant co-occurrence. This finding was consistent across cancer classes and was confirmed on an independent comparative genomic hybridization dataset of cancer samples. The results of our analysis are available for further investigation via an accompanying website.

**Conclusions:**

The broad generality and the intricate characteristics of the dichotomy of aneuploidy, ranging across numerous tumor classes, are revealed here rigorously for the first time using statistical analyses of large-scale datasets. Our finding suggests that aneuploid cancer cells may use extra chromosome gain or loss events to restore a balance in their altered protein ratios, needed for maintaining their cellular fitness.

## Background

Most cancer genomes undergo large scale alterations that dramatically alter their content and structure [1]. This phenomenon of genomic instability is responsible for the wide repertoire of chromosomal aberrations observed in cancer genomes. While the roles of most aberrations in the carcinogenesis process remain to be determined, the common perception [2] is that some of these aberrations are functionally important to the initiation and growth of cancer (drivers), while others merely represent random somatic changes that carry no selective advantage to the cancer cell (passengers). The identification of strong associations among aberrations - that is, associations that are observed significantly more than expected by chance - may help in the detection of driver aberrations or point to mechanisms that promote the selection of certain aberrations. As data on chromosomal aberrations in cancer accumulate, the detection of such strong associations can become more accurate and powerful.

Following the four-step model for colorectal cancer evolution suggested by Vogelstein and colleagues [3,4], several computational methods were developed for reconstructing common evolutionary paths of chromosomal aberrations in specific cancers. Some of these methods used tree models [5-7], later extended to acyclic networks [8-10]. These evolutionary models enable recognition of aberrations that occur at early stages of cancer; often referred to as 'primary', they are suspected of being cancer drivers. As these methods were designed to analyze samples from the same cancer type, they were applied to relatively small datasets, each containing a few hundred samples. More recently, a statistical method named GISTIC [11] was developed for identifying copy-number aberrations whose frequency and amplitude are higher than expected. This method was used in [12] for studying copy number alterations appearing at significant frequencies across several cancer types. In another recent study [13], profiles of frequent deletion events were analyzed in order to distinguish between driver and passenger deletion events. The latter two studies focus on copy number alterations of focal regions, derived by high-resolution techniques, from a heterogeneous pool of cancers with several hundred to a few thousand samples.

The Mitelman Database [14] is the largest depository of chromosomal aberrations in cancer. Although the aberrations are described using karyotypes of low resolution, these methods are widely used, notably in hospital labs, where the database is the leading source of information for clinicians who diagnose and treat cancer. The large number of samples in the database makes it ideal for statistical analyses, which are capable of overcoming random errors. In this study we present the results of large-scale analysis of chromosomal aberrations from over 15,000 karyotypes of the Mitelman Database. By exploiting the huge number of karyotypes, reconstructing the aberrations in them, and developing appropriate statistical tests, we were able to recognize significant cross-cancer associations among aberrations and to identify correlations among tumor types.

Most observed alterations include chromosome gains/losses and translocations. As translocations directly affect a small number of genes, the role of many translocations in cancer causation has become much clearer over the years [15]. Chromosome gains and losses, on the other hand, are broad alterations affecting numerous genes whose significance to the carcinogenesis process is much less understood. In this study we demonstrate strong associations involving chromosome gain and loss aberrations, suggesting selection preferences for aneuploid cells.

The results of our analysis, including the computed associations and links to their underlying karyotypes, are publicly available for further investigation via our website [16]. Each karyotype is linked to its original record in the Mitelman Database, thus allowing browsing of its full details. To the best of our knowledge, this is the first resource providing statistical results on such associations among cancer karyotypes.

## Results

**Figure 1:**
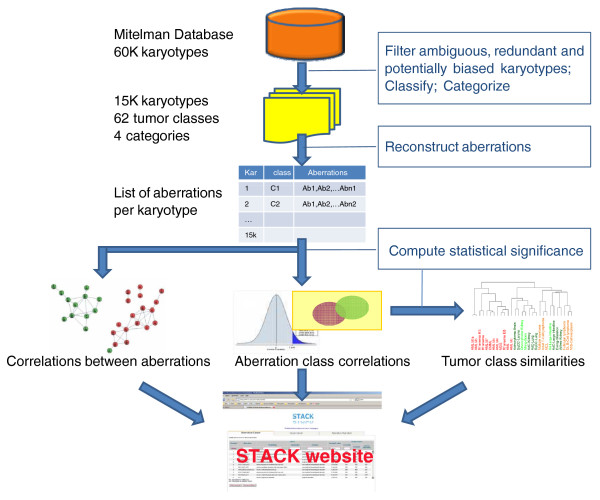
**Overview of karyotype analysis and the STACK website**. A large fraction of the karyotypes in the Mitelman Database was removed to avoid potential bias in the analysis. These included partially characterized karyotypes, multiple karyotypes from the same individual, and karyotypes that were not randomly selected in the original report. Tumor type and location were used to classify karyotypes into tumor classes, and classes with small representation (< 50 karyotypes) were removed from the dataset. An algorithm was used to reconstruct the set of aberrations leading to each remaining karyotype. Three types of statistical correlations were computed: aberration co-occurrence, association between class and aberration, and class similarity (based on their common aberrations). All computed correlations, with their *P*-values, are available for further investigation via our website [16] and are directly linked to the full description of the relevant karyotypes in the Mitelman Database. Repeating the analysis without filtering ambiguities and selected karyotypes (yielding 42,763 karyotypes, 83% of the Mitelman Database) led to essentially the same conclusions.

Figure 1 summarizes our karyotype analysis. Starting from 59,579 karyotypes in the Mitelman Database (November 2009 version), we used only 34,107 karyotypes that were annotated as unselected in order to avoid over- or under-estimation of aberration frequencies due to biases in sample selection [17]. We then filtered out any partially characterized or possibly redundant karyotypes, as well as karyotypes that were not near diploid. Tumor classes were defined according to tissue morphology and organ. Karyotypes belonging to classes with small representation (< 50 karyotypes) in the remaining dataset were omitted from analysis, resulting in a total of 62 classes and 15,445 karyotypes (Table 1).

**Table 1:** Tumor classes and categories in the dataset

Class	Number of karyotypes
**Benign solid tumors**	**1,567**
Ad-large intestine	100
Ad-salivary gland	191
Ad-thyroid	66
Benign-breast	69
Ch hamartoma-lung	99
Leiomyoma-uterus	214
Lipoma-ST	269
Mnng-brain	508
Oncocytoma-kidney	51
	
**Non-lymphoid hematological disorders**	**6,913**
AML	1,026
AML M0	144
AML M1	315
AML M2	776
AML M3	525
AML M4	621
AML M5	266
AML M5a	52
AML M6	133
AML M7	168
BBL	137
CMD	69
CML at	409
CML t(9;22)	808
CMML	147
Id myelofibrosis	115
JML	50
MDS	187
Polycythemia vera	166
Rf anemia	374
Rf anemia EB	344
Rf anemia RS	81
	
**Lymphoid disorders**	**4,411**
ALL	1,817
Adult T-cell lymphoma	64
Ang T-cell lymphoma	71
Burkitt lymphoma	248
CLL	884
DL B-cell lymphoma	197
Follicular lymphoma	274
HCL	57
M B-cell neoplasm	166
MCL	78
Multiple myeloma	385
Per T-cell lymphoma	62
SMZ B-cell lymphoma	108
	
**Malignant solid tumors**	**2,554**
AdC-breast	323
AdC-kidney	610
AdC-large intestine	125
AdC-ovary	56
AdC-prostate	124
AdC-thyroid	84
AdC-uterus	62
Astrocytoma-brain^a^	234
BCC-skin	87
Ewing-skeleton	181
Giant cell-skeleton	60
Hpblastoma-liver	65
Liposarcoma M-ST	59
Melanoma-eye	72
SqCC-larynx	58
SqCC-lung	64
Synovial sarcoma-ST	58
Wilms-kidney	232

Ad, adenoma; Adc, adenocarcinoma; ALL, acute lymphoblastic leukemia; AML, acute myeloid leukemia; Ang, angioimmunoblastic; BBL, bilineage or biphenotypic leukemia; BCC, basal cell carcinoma; Ch hamartoma, chondroid hamartoma; CLL, chronic lymphocytic leukemia; CMD, chronic myeloproliferative disorder; CML, chronic myeloid leukemia; CML at, CML aberrant translocation; CMML, chronic myelomonocytic leukemia; DL, diffuse large; HCL, hairy cell leukemia; Hpblastoma, Hepatoblastoma; Id, idiopathic; JML, juvenile myelomonocytic leukemia; Liposarcoma M, liposarcoma myxoid/round cell; M B-cell, mature B cell; MCL, mantle cell lymphoma; MDS, myelodysplastic syndrome; Mnng, meningioma; Per, peripheral; Rf anemia, refractory anemia; Rf anemia EB, refractory anemia with excess of blasts; Rf anemia RS, refractory anemia with ringed sideroblasts; SMZ, splenic marginal zone; SqCC, squamous cell carcinoma; ST, soft tissue.

^a^Astrocytoma grade III-IV

Each class was assigned to one of four sets: lymphoid disorders, non-lymphoid hematological disorders, benign solid tumors, and malignant solid tumors (Table 1). Due to its higher rate of successful karyotypic analyses, the group of hematological disorders dominated our dataset, with 11,324 (73%) karyotypes, of which 6,913 (45%) belong to non-lymphoid hematological disorders. We computed for each karyotype a set of most likely aberrations involved in its formation using 11 types of chromosomal rearrangement, deletion, and duplication events (Materials and methods; Additional file 1). Of those events, chromosome gain/loss and translocation were most frequent (Additional file 2). An aberration was identified by its causing event and the chromosomal locations it involved. For example, the translocation involving bands 9q34 and 22q11 was identified by t(9;22)(q34;q11), following the ISCN terminology [18].

### Aberrations characteristic of specific tumor classes

The karyotypes in our dataset contained 5,179 distinct aberrations, including all possible chromosome gains and losses. We computed the significance of the correlation of each aberration-class pair using the hypergeometric test. Out of 9,208 distinct observed aberration-class pairs, 1,705 were found to be significantly correlated at a false discovery rate (FDR) of 5% (website). These correlations encompassed all 62 tumor classes in our dataset, involving 1,360 distinct aberrations, where more than half of these correlations (907, 53%) involved translocations. Many of these strong correlations, notably the ones involving translocations, have been well documented in the literature: for example, t(9;22) in chronic myelogenous leukemia [19] and t(11;22) in Ewing sarcoma [20]. This supports the use of our dataset as a valid sample of karyotypes from the considered classes, as well as the soundness of our results.

### Two distinct paths to aneuploidy?

**Figure 2:**
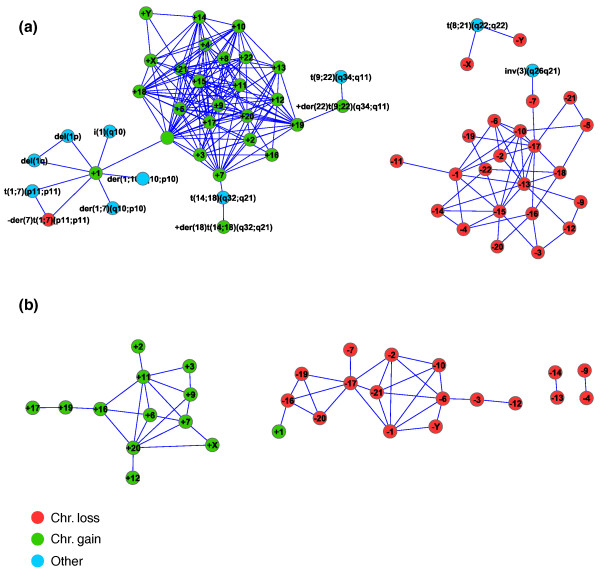
**Highly co-occurring aberration pairs**. Highly co-occurring aberrations in the entire karyotype dataset are connected by lines. Aberrations that are involved only in expected links (for example, a link between a translocation and a gain/loss of one of its derivative chromosomes; a link between two (two-break) translocations originating from one three-break [18] rearrangement) are not shown. For explanations of aberration names, see Additional file 1. **(a) **Highly co-occurring pairs in the Mitelman Database karyotypes (links are significant at *P *< 0.05, after Bonferroni correction). **(b) **Highly co-occurring pairs in the comparative genomic hybridization dataset (links are significant at FDR 5%). The only gain-loss link is (+1, -16), which has the second worst (that is, highest) *P*-value among the 47 pairs that passed the FDR 5% criterion. The figure was drawn using Cytoscape [40].

We now address a question that can be answered only by complex analysis of a large database: which aberrations tend to co-occur? We seek pairs of aberrations that appear together in karyotypes significantly more than expected by chance. Such associations may reveal either cooperation between different oncogenic events or common mechanisms creating chromosomal aberrations. To answer this question we tested the significance of co-occurrence for 7,202 aberration pairs in our dataset that satisfied the following two conditions: each aberration appeared in at least ten karyotypes, and the pair appeared together in at least one karyotype. We first filtered pairs with hypergeometric *P*-value > 0.001, leaving 623 pairs whose significance was further evaluated by a permutation test. Our analysis yielded 218 significantly co-occurring aberration pairs (*P *< 0.05, after Bonferroni correction), of which 154 (71%) were chromosome gain pairs, and 47 (22%) were chromosome loss pairs. The induced network split clearly into two disjoint parts: one dominated by chromosome gains and one by chromosome losses (Figure 2a). We carried out the same analysis separately for lymphoid disorders, non-lymphoid hematological disorders, solid tumors, and carcinomas (Figure S2a-d in Additional file 3). Each of these groups showed the same clear strong co-occurrence of specific gain-gain and loss-loss pairs, with almost no cases of significant co-occurrence for any mixed gain-loss pairs. We also detected the trisomy of 1q [21], which appeared in all tumor categories in the associations involving gain of chromosome 1 (Figure 2a; Figure S2a-d in Additional file 3).

We repeated this test on an extended dataset of 42,763 karyotypes, which included selected and partially characterized karyotypes (omitting non-characterized fragments). The two disjoint clusters of chromosome gains and losses are still clearly evident in the obtained results (Figure S2e in Additional file 3). The major observed change in the results is the addition of many new significant associations that involve aberrations other than chromosome gains and losses. This addition is explained by the growth in the amount of data, which increased the power of the statistical test, allowing it to uncover weaker associations. To confirm this, we examined an extended set of significant co-occurring aberrations (FDR 5%) in the original (filtered) dataset and obtained essentially the same results (not shown).

To test our result on independent data obtained using a different technology, we used data from comparative genomic hybridization (CGH), a laboratory method to measure gains and losses in the copy number of chromosomal regions in tumor cells. We analyzed an independent dataset of 1,084 samples obtained by CGH, downloaded from the NCI and NCBI's SKY/M-FISH and CGH database (16 March 2009 version). This database contains CGH records contributed by molecular cytogeneticists for open investigation. Each sample was assigned a corresponding set of whole chromosome gain/loss aberrations, yielding 648 (60%) samples with non-empty aberration sets. Using a permutation test similar to the one used for karyotype data (Materials and methods), we computed a *P*-value for the co-occurrences of specific aberration pairs in the CGH dataset. Out of 856 distinct co-occurring aberration pairs, 47 were significantly co-occurring at a FDR of 5%. The picture obtained with these pairs (Figure 2b) is strikingly similar to that produced by the karyotype data. This reaffirms our observation that the progression of aneuploidy in cancer is driven by either multiple chromosomal gains or multiple chromosomal losses.

### A similarity map of tumor classes

Which tumor classes have highly similar aberrations? Using the set of significant (FDR 5%) aberration-class correlations, we assessed the statistical significance of the overlap in aberrations for every pair of tumor classes. Of all 1,891 possible class pairs, 56 were found to significantly share common aberrations at a FDR of 5% (Figure S3a1 in Additional file 4). Considering benign and malignant solid tumors as one category, all but three (53, 95%) of these pairs belong to the same category, with two of the three exceptions linking between lymphoid disorders and (malignant) solid tumors. We repeated the analysis, expanding the set of correlative aberrations by considering also weaker correlations with (uncorrected) *P*-value < 0.05. The results show a remarkably similar partition, with 86 significant class pairs (FDR 5%), forming three distinct clusters, with only six links between the sets of lymphoid disorders and solid tumors (Figure S3a2 in Additional file 4). The fact that the categories were very well separated serves as confirmation of the data and of our methodology.

**Figure 3:**
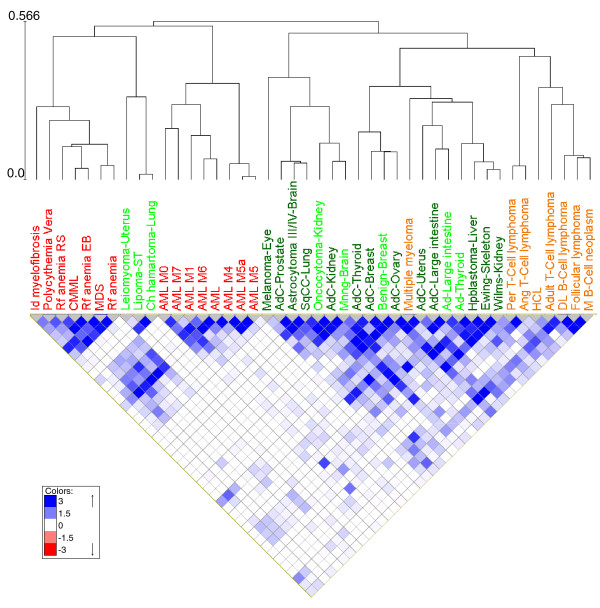
**Hierarchical clustering of classes based on class similarity in sharing common aberrations**. The square at the intersection of each two diagonals shows the similarity of their classes as measured by the aberrations associated with them (Materials and methods). (An aberration was associated with a tumor class if the correlation had a (uncorrected) *P*-value < 0.05.) Names of cancer classes are colored as follows: orange, lymphoid disorders; red, non-lymphoid hematological disorders; light green, benign solid tumors; dark green, malignant solid tumors. Classes that showed no significant similarity to any other class at FDR 5% were not included in the clustering.

For more in-depth study of similarity among classes, we defined a similarity measure between classes based on the significance of their common aberrations (Materials and methods) and used it to hierarchically cluster the classes (Figure 3). As before, classes of the three sets - non-lymphoid-hematological disorders, lymphoid disorders and solid tumors - clustered separately. A deeper look into each cluster (Figure 3) revealed that many closely clustered classes were histologically related. For example: diffuse large B-cell lymphoma, follicular lymphoma, and mature B-cell neoplasm (B-cell lymphomas); adenoma and adenocarcinoma in the large intestine; and AML M5 and AML M5a. The correlated aberrations shared by two similar classes can be viewed through our website. One of the interesting results was the close proximity of three embryonic cancers: Wilms' tumor (kidney), Ewing sarcoma (skeleton) and Hepatoblastoma (liver).

### The website

All the associations described above can be viewed via the website [16], which contains summary tables for the different types of associations: aberration-class, class-class, and aberration-aberration. Table rows can be filtered textually and numerically, allowing investigations of associations for a specific group of cancer types, a set of aberrations of interest, or both. For example, the user can view all aberrations whose correlation with a certain tumor class is below some specified *P*-value. Alternatively, all aberrations significantly co-occurring with a specified aberration can be examined, with their *P*-values. For aberration-class and aberration-aberration associations, researchers can examine the karyotypes that led to these associations, where each karyotype is linked to its corresponding record in the Mitelman Database website.

To demonstrate the utility of the website, we focused on hyperdiploid multiple myeloma (H-MM), a subtype of multiple myeloma (MM) with better prognosis, characterized by having 48 to 74 chromosomes [22-24]. Our dataset included 385 MM karyotypes, 110 (29%) of which were hyperdiploid. H-MM is associated with recurrent gains of chromosomes 3, 5, 7, 9, 11, 15 and 19 [22]. Indeed, the website's class-aberration table, filtered for MM associations, confirmed this observation: +3, +5, +9, +11, +15, and +19 were the aberrations most associated with MM, and the 142 karyotypes involved in these associations spanned all H-MM karyotypes (hyper-geometric *P *< 1E-76). Chng *et al*. [25] suggested a FISH-based trisomy index test for identifying H-MM, employing probes for chromosomes 9, 11 and 15, and designating a tested MM cell as H-MM if it contains two or more trisomies in these chromosomes (see Materials and methods). They reported specificity of 0.98 and sensitivity of 0.69 for that index. The corresponding F-score (a measure combining sensitivity and specificity; see Materials and methods) was 0.8. We analyzed the 385 MM karyotypes in the same fashion as [25]; the criterion of any two trisomies in 9, 15, 19 was best with specificity 0.996 and sensitivity 0.88 (F-score 0.93). In fact, the same combination has the highest F-score on the data from [25] as well (0.83). Thus, the criterion of two or more trisomies of chromosomes 9, 15, 19 should be considered for identifying H-MM.

## Discussion

In this study we computationally analyzed a large number of cancer karyotypes from the Mitelman Database, the largest available compendium of cancer karyotypes. Based on statistical analysis of more than 15,000 karyotypes, our results provide strong additional evidence for the non-randomness of many chromosomal aberrations in cancer. Our approach is validated by the demonstration of known relationships, including associations between specific aberrations and specific tumor types, and similarities among certain tumors (for example, adenoma and adenocarcinoma of the large intestines). More importantly, the analysis led to new discoveries, most notably that chromosomal aneuploidy tends to consist of either a pattern of chromosomal gains or a pattern of chromosomal losses. This discovery was verified on an independent CGH database. A similar tendency was observed by Höglund *et al*. [9] for a small number of specific solid cancers. The karyotypic evolution models of [9] contained two converging paths, one dominated by gains of chromosomal fragments and the other by losses. To the best of our knowledge, our results provide the first rigorous demonstration of this widespread association within chromosomal aneuploidy in cancer cells.

To avoid ambiguities and reduce potential biases in the results, we excluded from our dataset karyotypes that were not random samples (that is, reported because of a specific/unusual karyotypic feature), and those with missing information. Inclusion of partially characterized karyotypes (omitting non-characterized fragments) and karyotypes marked as selected (that is, non-random samples) increased the number of karyotypes to 42,763 (83% of the Mitelman Database). The results with that set closely match those reported here (Figures S2e in Additional file 3 and S3b in Additional file 4), indicating the robustness of both the results and our statistical methods.

Chromosome gains/losses and translocations were the most abundant aberrations in our dataset. While many translocations were shown to contribute to carcinogenesis, the role of chromosomal aneuploidy in cancer has been debated for almost a century. Aneuploidy generally interferes with cellular growth and proliferation, but is frequently associated with the disease of uncontrolled proliferation, cancer. In yeast, aneuploid cells show a transcriptional response similar to that described in yeast cells grown under many different stress conditions [26]. As protein expression levels largely scale with chromosome copy numbers [27], this may reflect the aneuploid cell's effort to reestablish protein stoichiometry [26]. The detrimental role of accumulated proteins in aneuploid cells is supported by a recent report demonstrating that mutations accelerating protein degradation increased the tolerance for anueploidy [28]

These observations may explain the striking chromosome gain/loss dichotomy that we observed and suggest a partial explanation for the following conundrum: a germline or experimentally acquired single chromosome gain/loss is usually detrimental, both at the cellular and the organism levels, while the abundance of chromosome gains/losses in cancer cells implies that aneuploidy is beneficial, or at least not harmful, to their vitality [29-33]. As most chromosomes contain dosage-sensitive genes, the strong gain-gain and loss-loss correlations may imply a mechanism for balancing the ratios of proteins that function in complexes. Such balancing may be required to protect the cancer cell from the detrimental effects of partially assembled protein complexes or free subunits by molecular chaperones caused by prior chromosome gain/loss events.

An alternative explanation for these observations is that chromosomal gains and losses are caused by different mechanisms of genomic instability. This is less likely, however, as it implies that defects in the mitotic checkpoint result in non-random distribution of the aneuploidy chromosomes between two daughter cells. There are no experimental data to support that hypothesis. A third possible explanation is that the correlation of gains with other gains and losses with other losses is driven by catastrophically failed mitoses, where many chromosomes fail to separate during anaphase. In this scenario one daughter cell wound up with many more, and the other with many fewer chromosomes. However, this scenario does not explain why many specific chromosome pairs are significantly co-gained/co-lost, even when the statistical test is corrected for chromosome gain-loss dichotomy (results not shown). Additional experimental data are needed to substantiate or refute these hypotheses.

Interestingly, gain-gain correlations are more prevalent and more significant than loss-loss co-occurrences (compare Figure 2 and the website). There may be two explanations for why gains of chromosome pairs are more common than losses. The first is simply mathematical: trisomy means 30% more dosage for a set of genes, while a loss implies a more dramatic 50% drop. The second is experimental - Rancati *et al*. [34] have shown that the higher the ploidy the better the adaptation to aneuploidy is. Hence, gains of multiple chromosomes may be advantageous in the evolution of human cancer karyotypes.

One limitation of the use of the Mitelman Database is the very low resolution of the karyotypes, disallowing identification of low-level and focal events. On the other hand, the huge number of karyotypes allowed us to carry out rigorous statistical analysis on a very large scale. Another limitation is its inherent bias towards hematological cancers. However, the number of solid karyotypes in the database is still substantial, and allowed us to obtain results on class similarity among solid cancers (Figure 3). Moreover, the results on aberration co-occurrence tendency were similar using the full data (Figure 2) and the solid karyotypes only (Figure S2c in Additional file 3). Cytogenetic techniques are still widely used in cancer studies, and have some advantages over current high-resolution techniques. Cytogentic methods allow distinguishing between different clones that co-exist in a cancer sample, and are often used in verifying the existence of specific aberrations. We emphasize that in our analysis we analyzed all types of aberrations identifiable by cytogentic techniques, including translocations, iso-chromosomes, partial deletions, and more. Nevertheless, the strongest associations we revealed among aberrations involved mainly whole chromosome gains and losses, most likely since other aberrations (for example, specific translocations, or deletions) are less common and more difficult to detect using cytogentic techniques.

The methodologies developed in this study can be used on other large datasets describing genetic events. As high-resolution genetic information on tumors (for example, from array-CGH and deep sequencing) accumulates, similar analysis can be applied to it. For example, Beroukhim *et al*. [12] demonstrated that a large majority of somatic focal copy-number alterations identified in individual cancer types are present in several cancer types. Our method can be used to assess whether common somatic focal copy-number alterations tend to be shared by related cancers, as has been the case for cytogentic aberrations. The main challenges in adapting our methods for array-based data are assigning each sample with a set of aberrations (aka 'aberration calling'), and handling intersecting aberrations (for example, two deletions with overlapping segments). Another major difficulty in uncovering strong associations in cancer data is the requirement for a large number of cancer samples. To obtain a large dataset, we performed pooled analysis of heterogeneous cancer samples, similarly to [12,13]. Pooled analysis has the potential of revealing associations possibly pertinent to common cellular mechanisms shared by different cancer types. Recent examples include: cancer-related genes hosted in highly frequent copy-number alterations in cross-cancer data [12], structural signatures of driver/passenger homozygous deletions [13]; and the whole chromosome gain/loss dichotomy phenomenon reported here.

Finally, our website can be useful both for additional global investigations like those reported here and for in-depth analysis of individual associations.

## Conclusions

Cancer is a common name for many different diseases: there is large variability among different cancers, and even among cancers of the same morphological and topographical origin. Nevertheless, different cancers may share similar mechanisms. Analyzing a heterogeneous set of cancers has the potential of uncovering patterns that are related to such common mechanisms. In this study we performed a large-scale analysis of karyotypes from heterogeneous cancer samples. We show that many aberrations, including some whole chromosome gains and losses, are highly specific to certain cancers. Other aberrations exhibiting weaker specificity were shown to be shared among cancers of related morphology. The investigation of aberration pairs revealed a striking non-random, cross-cancer pattern of aneuploidy, where whole chromosome gains are associated with other gains and whole chromosome losses are associated with other losses. Despite being very common, the role of aneuploidy in cancer initiation or progression is unclear, but one explanation of the non-random pattern of aneuploidy that we have found and quantified is that it is necessary for a clonal growth advantage. We hope that this finding will lead to a better understanding of the mechanisms that allow cancer cells to balance the harms with the potential growth advantage caused by aneuploidy.

## Materials and methods

### Karyotype selection and analysis

Starting from all 59,759 karyotypes present in the Mitelman Database on 17 November 2009, we carried out several aggressive filtering steps aiming to reduce ambiguity and avoid any possible bias (see Additional file 5 for the full details). Briefly, we evaluated all 34,107 karyotypes marked as unselected (that is, chosen in a non-biased manner). Karyotypes were parsed using the CyDAS ISCN parser [35], and any karyotype detected as invalid during the parsing was excluded, leaving 29,911 (88%) valid karyotypes. We filtered all karyotypes that are not well-defined. For a multiclonal karyotype, we avoided dependency between its karyotypes by choosing only the first well-defined karyotype it contained. In case of multiple karyotypes from the same patient ('case' in the Mitelman Database), only one karyotype was taken into account. To avoid potential biases in chromosome gain/loss aberrations, we excluded any karyotype that was not near-diploid (that is, we omitted karyotypes whose total chromosome number was <35 or >57). Altogether, 18,813 karyotypes were selected for analysis.

### Aberration reconstruction

We previously identified 11 frequent chromosomal events in tumor karyotypes (chromosome gain/loss, translocation, deletion, duplication and more; Additional file 1), and developed an algorithm for reconstructing a most plausible set of these events leading to a given karyotype [36]. Briefly, our algorithm mimics the intuitive way a researcher would perform this task manually: starting with the cancer karyotypes, the algorithm selects the simplest and most evident step of 'undoing' one event at a time, bringing the karyotype closer to the normal one. We applied the algorithm to all relevant karyotypes from the Mitelman Database, obtaining unambiguous reconstruction in 99% (18,600) of the karyotypes. We recorded each karyotype's set of aberrations, where an 'aberration' is defined by an event and the chromosomal locations involved. See Additional file 5 for further details.

### Karyotype classification

We classified karyotypes by their tissue morphology and topography as specified in the Mitelman Database. To permit robust statistical analysis, we omitted all karyotypes whose class had less than 50 karyotypes. Our final dataset contained 15,445 karyotypes.

### Comparative genomic hybridization data

To validate our results for co-occurrence of chromosome gains and losses, which were obtained using karyotype data, we searched for an alternative independent dataset. We used the NCBI's SKY/M-FISH and CGH database [37] (16 March 2009 version), consisting of 1,084 records. Every record has a list of chromosomal segments with abnormal copy number, each classified as a gain or a loss, and the header of the record contains information on the cancer tissue. As most tumor classes in this dataset were relatively small, we ignored the histological classification. For each record we derived chromosome gain/loss aberrations in the following manner: every gained (lost) chromosomal fragment that spanned the centromere was considered a whole chromosome gain (loss). Gain/loss aberrations that were internal to a chromosome arm (that is, not spanning the centromere) were ignored.

### Computing *P*-values for aberration-class correlations

For an aberration *Ab *and a tumor class *C*, we calculated the significance of the enrichment of karyotypes with *Ab *in *C *using the hypergeometric test.

### Computing *P*-values for classes sharing common aberrations

We say that an aberration *Ab *is *t*-correlative to a tumor class *C *if the enrichment of karyotypes with *Ab *in *C *had a hypergeometric *P*-value ≤ *t*. For a fixed *t*, we developed the following method for evaluating the significance of shared aberrations between tumor classes. We constructed a binary matrix *M_t _*whose rows and columns correspond to aberrations and classes, respectively. We set *M_t_*[*Ab*,*C*] = 1 if *Ab *is *t*-correlative to *C*, and otherwise *M_t_*[*Ab*,*C*] = 0. For *t *= 0.05, the maximal *t *used in our analysis, the matrix *M_t _*was already quite sparse, with less than 2% of the values being 1.

For two classes, *C1 *and *C2*, we computed a *P*-value for their number of shared events as follows. Let *n_t.C1, C2 _*be the number of *t*-correlative aberrations that *C1 *and *C2 *share. More formally:

For every pair of classes, *C1 *and *C2*, we estimated the probability of having at least *n_t, C1, C2 _t*-correlative aberrations by chance by sampling *N *= 10^7 ^randomized permutations of *M_t _*that preserve row and column sums. Every such permutation corresponds to an assignment of aberrations to tumor classes that keeps the general properties of the original data: aberrations that occur in few (or many) cancer classes remain so, and tumor classes that had many (or few) correlative aberrations preserve this property. The randomization is done by a long sequence of edge swaps [38]. The *P*-value for *C1 *and *C2 *is defined as *r*(*C1*,*C2*,*N*,*t*)/*N*, where *r*(*C1*,*C2*,*N*,*t*) is the total number of *M_t _*permutations in which the number of aberrations that *C1 *and *C2 *share is ≥ *n_t, C1, C2_*. In case *r*(*C1*,*C2*,*N*,*t*) = 0, we defined the *P*-value to be 1/*N*. Therefore, the minimal *P*-value we could achieve was 10^-7^.

### Hierarchical clustering of classes

We performed average-linkage hierarchical clustering of the classes using the Expander software package [39]. The similarity measure between classes was defined as follows. We first built a symmetric matrix, *S*, satisfying *S*[*C_1_*,*C_2_*] = -*log*(*p*), where *p *is the *P*-value described above for the significance of the number of *t*-correlative aberrations that *C_1 _*and *C_2 _*share, *n_t.C1, C2_*. For each class *C*, we set *S*[*C*,*C*] = *log*(*N*), where *N *= 10^7 ^as above. The similarity between classes was now defined as the Pearson correlation between their rows of *S*. Classes showing no significant similarity at FDR 5% to any other class were removed from this analysis.

### Computing *P*-values for co-occurring aberration pairs

For two aberrations, *Ab1 *and *Ab2*, let *n*(*Ab1*, *Ab2*) be the total number of karyotypes that contain both aberrations. We estimated the significance of *n*(*Ab1*, *Ab2*) for all pairs of distinct aberrations using a permutation test (similar to the one described above) as follows. We constructed a binary matrix, *M*, whose rows correspond to aberrations that occur in at least ten karyotypes, and columns to karyotypes. For an aberration *Ab *and karyotype *K*, we set *M*[*Ab*,*K*] = 1 if *K *contained *Ab*, and *M*[*Ab*,*K*] = 0 otherwise. We randomly sampled permutations of *M *that preserved row and column sums. Thus, each permutation corresponds to a random set of karyotypes with the same distributions of (i) number of aberrations per karyotype, and (ii) number of karyotypes per aberration. Moreover, to account for the different distributions of aberrations within each tumor class, the sampled permutations were also required to preserve (sub-)row sum corresponding to each class. We enhanced the performance of this test by filtering aberration pairs whose hypergeometric test *P*-value was > 0.001, and removing from *M *any aberration that did not appear in the remaining pairs.

We performed a similar test for the CGH dataset, but since it was smaller in size we used all aberrations (that is, irrespective of the number of samples in which they were found), and without the step of filtering pairs by the hypergeometric test.

### Trisomy index test

To demonstrate the utility of our website, we used it to define a trisomy index test (TI-T), a test that uses specific trisomies (that is, chromsome gains) in order to distinguish between prognostically different subgroups of a certain disease. Similar to Chng *et al*. [25], we focused on H-MM, a subtype of MM. For a given TI-T, the sensitivity (respectively, specificity) was calculated as the percentage of H-MM (respectively, non-H-MM) karyotypes that are correctly identified as such by the TI-T. The positive predictive value (PPV) was calculated as the percentage of H-MM karyotypes among all karyotypes identified as H-MM by TI-T. The F-score was calculated as the harmonic mean of sensitivity and PPV: F = 2 × PPV × Sensitivity/(PPV + Sensitivity).

## Abbreviations

CGH: comparative genomic hybridization; FDR: false discovery rate; FISH: fluorescence *in situ *hybridization; H-MM: hyperdiploid multiple myeloma; MM: multiple myeloma; PPV: positive predictive value; TI-T: trisomy index test.

## Authors' contributions

RS and MOF designed research. MOF performed research and built the website. CL and MOF developed the statistical scores. MOF, RS, SI and LT analyzed and interpreted the data. MOF, RS and SI drafted the manuscript. All authors read and approved the final manuscript.

## Supplementary Material

Additional file 1 **Table S1**. Chromosomal events allowed in the reconstruction algorithm. Click here for file

Additional file 2 **Figure S1**. Event frequencies. Click here for file

Additional file 3 **Figure S2**. Highly co-occurring aberration pairs. Highly co-occurring aberrations (*P *< 0.05 after Bonferroni correction) are connected by lines. Aberrations that are involved only in expected links are not shown. See Additional file 1 for aberration name abbreviations. **(a) **Lymphoid disorders, **(b) **non-lymphoid hematological disorders, **(c) **solid tumors, **(d) **carcinomas, **(e) **all karyotypes. Results were obtained on a dataset that includes partially defined and selected karyotypes (83% of the Mitelman Database). Legend is as in Figure 2 for (a-d), and for (e) with the addition of light red and light green colors corresponding to partial deletions and partial duplications, respectively. Click here for file

Additional file 4 **Figure S3**. Tumor classes with similar common aberrations. **(a) **Tumor class pairs with significantly high numbers of common aberrations are connected by lines (FDR 5%). Aberrations assigned to tumor classes are: (a1) significantly correlated at FDR 5%, (a2) correlated with *P*-value < 0.05 (uncorrected). **(b) **Hierarchical clustering of classes based on class similarity in sharing common aberrations. Results were obtained with a dataset that includes partially defined and selected karyotypes (83% of the Mitelman Database). Legend is as in Figure 3. Click here for file

Additional file 5 **Text S1**. Description of the algorithm for reconstructing aberrations from karyotypes. Click here for file
