# Supplementary material for: Large-scale analysis of chromosomal aberrations in cancer karyotypes reveals two distinct paths to aneuploidy
Source: Genome Biol. 2011 Jun 29;12(6):R61. doi: 10.1186/gb-2011-12-6-r61 (PMC3218849; doi:10.1186/gb-2011-12-6-r61)
Supplement: Additional file 1 — Table S1. Chromosomal events allowed in the reconstruction algorithm. [file gb-2011-12-6-r61-S1.DOC]

Supplementary Table 1, **Chromosomal events allowed in the reconstruction algorithm**

| **#** | **Event  (aberration type)** | **Description** | **Notation (ISCN based)** | **Example** |
| --- | --- | --- | --- | --- |
| 1 | CHR_GAIN | Duplication of an existing chromosome | +*C* (chr. C is described using ISCN) | +1: gain of chromosome 1 |
| 2 | CHR_LOSS | Loss of an existing chromosome | -*C* | -X: loss of chromosome X |
| 3 | TRANSLOCATION | Exchange of ends between two chromosomes | t | t(9;22)(q34;q11): a translocation involving bands q34 and q11 in chromosomes 9 and 22 resp. |
| 4 | TAIL_DEL | Deletion of chromosome end | del | del(1q): a deletion of fragment in the q arm of chromosome 1 |
| 5 | IN_DEL | Deletion inside a chromosome |
| 6 | TAIL_DUP | Deletion of chromosome end + a duplication (and fusion) of another chromosome end instead. | der(..)t(..) | der(9)t(9;22)(q34;q11): A deletion of the end of the q-arm of chr. 9 (at band q34) and duplicating the q-arm of chr. 22 (at band q11). |
| 7 | ISOCHR | Isochromosome: Chromosome arm loss + duplication of remaining arm | i, idic, ider | i(1)(q10): the p arm (starting in q10- centromere) was lost, and the remaining q arm was duplicated , resulting in a chromosome with two identical arms |
| 8 | INVERSION | Inversion of a fragment within a chromosome | inv | inv(3)(q26q21): the fragment between the bands q26 and q21 was inverted in chr. 3 |
| 9 | PLOIDY_CHANGE | Multiplication of all chromosomes’ ploidy by the same factor | *x*n | 2n: diploid genome (normal) – no ploidy change. 4n: tetraploid genome |
| 10 | DICENTRIC | Deletion of two chromosome ends + fusion of the two new ends resulting in a dicentric chromosome | dic | dic(1;7)(p10;q10): composed of the q arm of chromosome 1 and the p arm of chromosome 7 (q10 and p10 correspond to centromeres) |
| 11 | TANDEM_DUP | Duplication in a chromosome, of the form ***abbc*** or ***ab(-b)c*** | dup | dup(1q): a tandem duplication in the q arm of chr. 1 |
| 12 | INSERTION | Excision of a fragment from one chromosome and insertion of it within another one | ins | ins(22;9)(q11;q34q34): excision of a fragment in band q34 of chr. 22 and insertion of it in band q11 of chr. 22 |
