# Supplementary figures and images for: Large-scale analysis of chromosomal aberrations in cancer karyotypes reveals two distinct paths to aneuploidy
Source: Genome Biol. 2011 Jun 29;12(6):R61. doi: 10.1186/gb-2011-12-6-r61 (PMC3218849; doi:10.1186/gb-2011-12-6-r61)

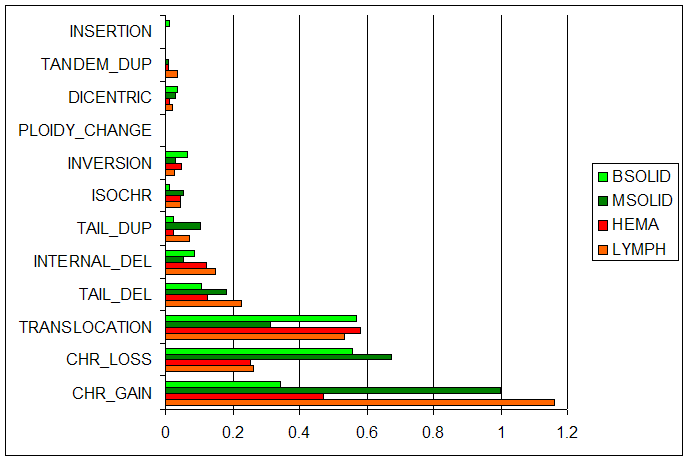

Supplement: Additional file 2 — Figure S1. Event frequencies. [file gb-2011-12-6-r61-S2.TIFF]

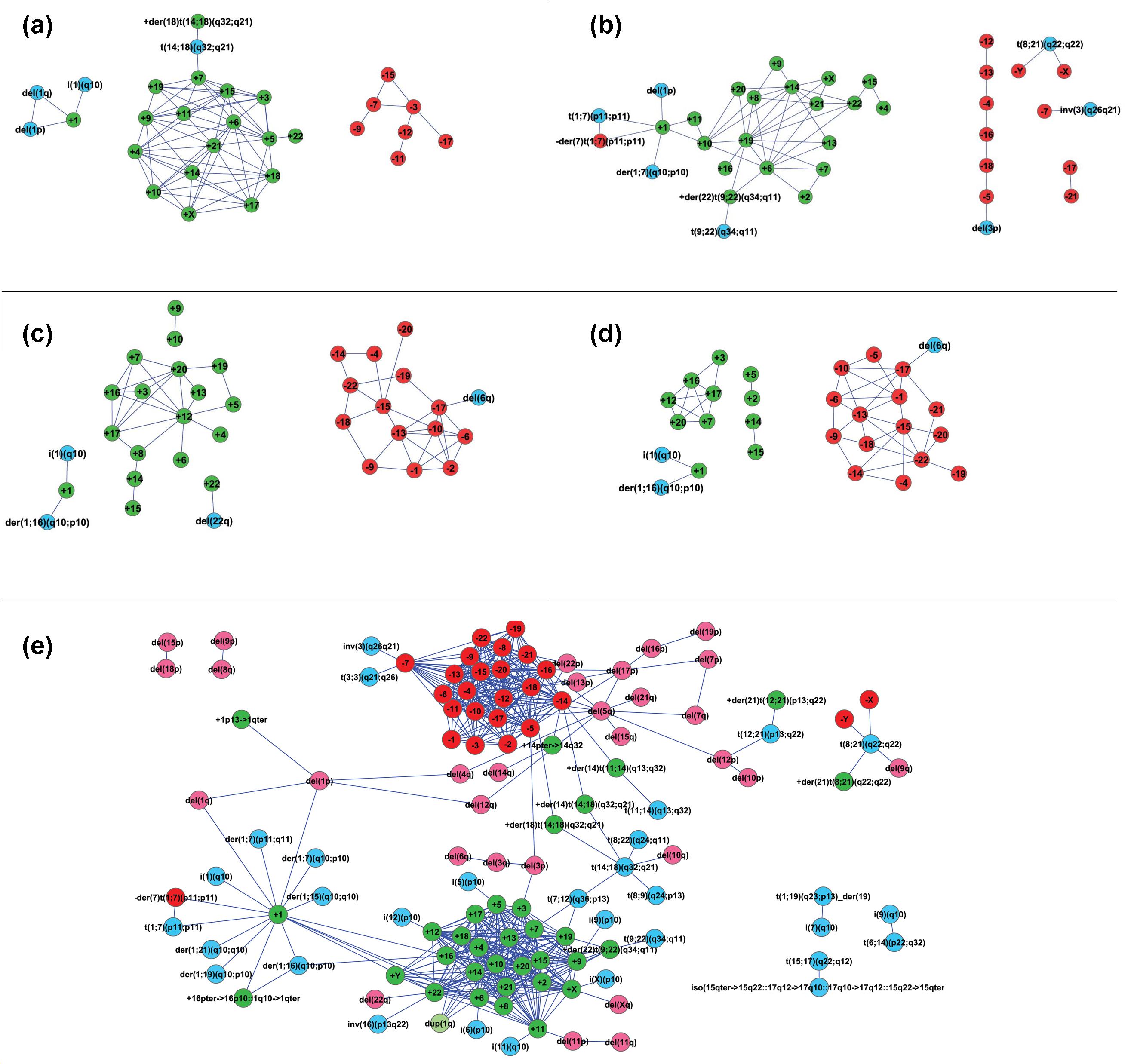

Supplement: Additional file 3 — Figure S2. Highly co-occurring aberration pairs. Highly co-occurring aberrations (P < 0.05 after Bonferroni correction) are connected by lines. Aberrations that are involved only in expected links are not shown. See Additional file 1 for aberration name abbreviations. (a) Lymphoid disorders, (b) non-lymphoid hematological disorders, (c) solid tumors, (d) carcinomas, (e) all karyotypes. Results were obtained on a dataset that includes partially defined and selected karyotypes (83% of the Mitelman Database). Legend is as in Figure 2 for (a-d), and for (e) with the addition of light red and light green colors corresponding to partial deletions and partial duplications, respectively. [file gb-2011-12-6-r61-S3.JPEG]

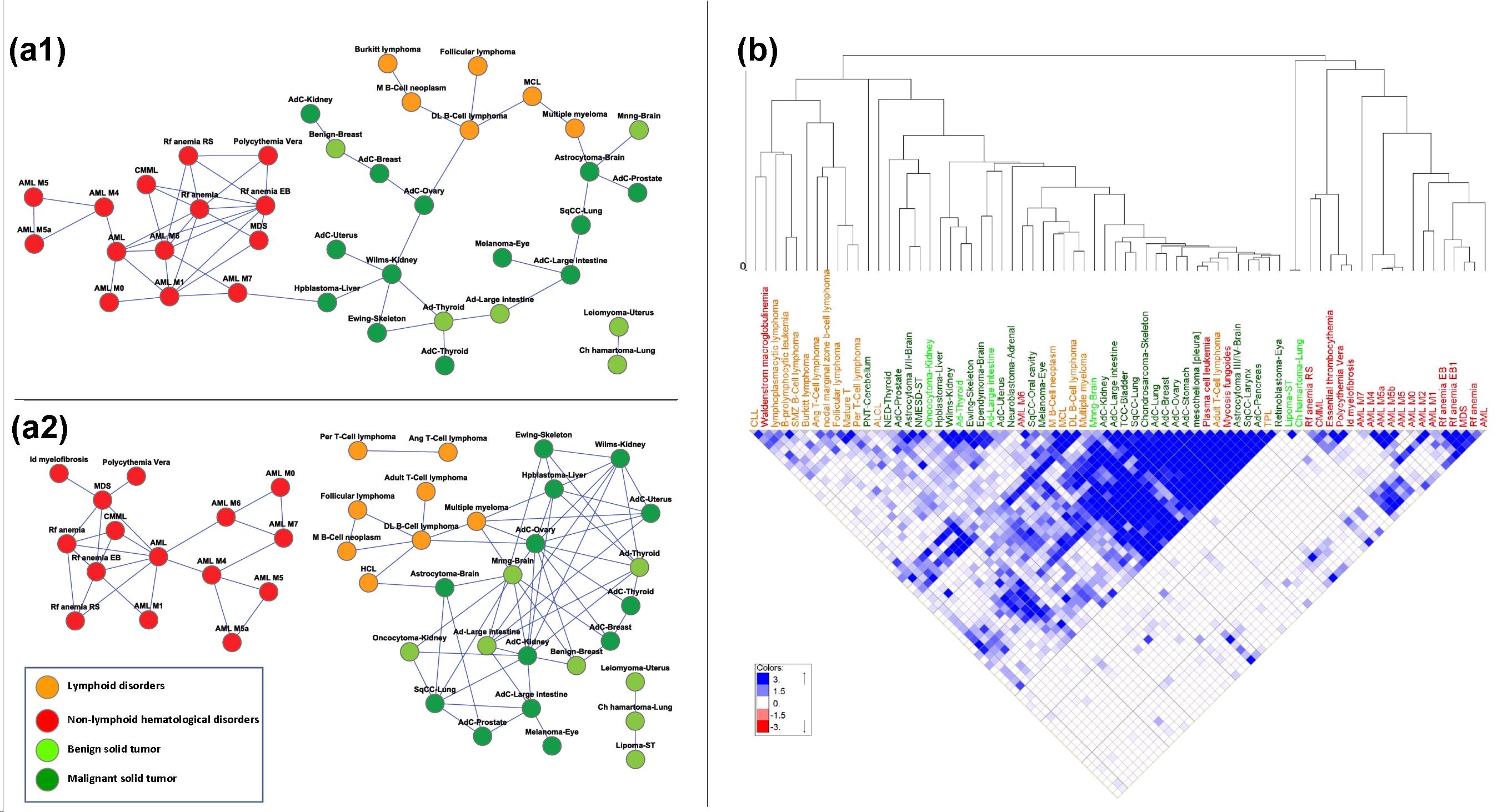

Supplement: Additional file 4 — Figure S3. Tumor classes with similar common aberrations. (a) Tumor class pairs with significantly high numbers of common aberrations are connected by lines (FDR 5%). Aberrations assigned to tumor classes are: (a1) significantly correlated at FDR 5%, (a2) correlated with P-value < 0.05 (uncorrected). (b) Hierarchical clustering of classes based on class similarity in sharing common aberrations. Results were obtained with a dataset that includes partially defined and selected karyotypes (83% of the Mitelman Database). Legend is as in Figure 3. [file gb-2011-12-6-r61-S4.JPEG]
