# Supplementary material for: Large-scale analysis of chromosomal aberrations in cancer karyotypes reveals two distinct paths to aneuploidy
Source: Genome Biol. 2011 Jun 29;12(6):R61. doi: 10.1186/gb-2011-12-6-r61 (PMC3218849; doi:10.1186/gb-2011-12-6-r61)
Supplement: Additional file 5 — Text S1. Description of the algorithm for reconstructing aberrations from karyotypes. [file gb-2011-12-6-r61-S5.DOC]

**Supplementary text for**

Ozery-Flato et al., Large-scale analysis of chromosomal aberrations in cancer karyotypes reveals two distinct paths to aneuploidy

**Karyotype selection and analysis**

We derived karyotypes for analysis by the following steps:

Step 1 - Exclusion of selected karyotypes: Out of 59,759 karyotypes present in the Mitelman database on November 17, 2009, we evaluated all 34,107 karyotypes marked as *unselected* (i.e. chosen in a non-biased manner).

Step 2 - Exclusion of partially defined karyotypes: Karyotypes were parsed using the CyDAS ISCN parser (14), and any karyotype detected as invalid during the parsing was excluded, leaving 29,911 (88%) valid karyotypes. We refer to a karyotype as *partially-defined* if it contains any of the following:

- Double minutes,
- Marker chromosomes,
- Ring chromosomes,
- Chromosomes with homogeneous staining regions (HSRs),
- Chromosomes with additional material of unknown origin,
- Approximated breakpoints, e.g. del(1)(q21~q24),
- Alternative interpretations of an aberration (designated by "or" symbol),
- “inc” symbol (which denotes “incomplete information”).

Question marks (?) indicating questionable identification of a chromosome or chromosome structure (e.g. del(1)(q?23)) were ignored (i.e. removed from the karyotype description).

Step 3 – Exclusion of dependent karyotypes: We refer to a karyotype as *multiclonal* if it is composed of several distinct karyotypes (separated by a dash “/” representing different subclones in the sample). Given a multiclonal karyotype, we avoided dependency between its karyotypes by choosing only the first well-defined karyotype it contained. In case of multiple karyotypes from the same patient (“case” in the Mitelman database), only one karyotype was taken into account.

Step 4 – exclusion of non-diploid karyotypes: To avoid potential biases in chromosome gain/loss aberrations, we excluded any karyotype that was not near-diploid (i.e., we omitted karyotypes whose total chromosome number was less than 35 or more than 57).

**Aberrations reconstruction**

We previously identified 11 frequent chromosomal events in tumor karyotypes (chromosome gain/loss, translocation, deletion, duplication and more, see Supplementary Table S1), and developed an algorithm for reconstructing a most plausible set of these events leading to a given karyotype (15, Supplementary Text). Briefly, our algorithm mimics the intuitive way a researcher would perform this task manually: Starting with the cancer karyotypes, the algorithm selects the simplest and most evident step of “undoing” one event at a time, bringing the karyotype closer to the normal one. Notably

- We present aberrations using ISCN-like notations. For example, +1 is the aberration resulting from a chromosome gain event on chromosome 1, and t(9;22)(q34;q11) is a translocation involving bands q34 and q11 on chromosomes 9 and 22, respectively.
- A chromosome gain / loss aberration always corresponds to a gain/loss of a *whole* chromosome.
- In order to find strong associations that involve similar (i.e. overlapping) partial duplications / deletions and to simplify the presentation of results, we reduced the resolution of corresponding *dup* and *del* aberrations to arm level. For example, the partial duplications, dup(17)(q21q25) and dup(17)(q12q12), are both presented as dup(17q).

We applied the algorithm to all relevant karyotypes from the Mitelman database, obtaining unambiguous reconstruction in 99% (18,600) of the karyotypes. We recorded each karyotype’s set of aberrations, where an *aberration* is defined by an event and the chromosomal locations involved.

**The Karyotype Sorting Algorithm**

We describe here an algorithm, which we call SKS (Simple Karyotype Sorter), for reconstructing a shortest sequence of rearrangement events (structural and numerical) from the normal karyotype to a given cancer karyotype. The sequence is a parsimonious explanation of the progression of rearrangements leading to the observed karyotype. This algorithm was originally described in [1]. We call this process sorting the karyotype. The SKS algorithm aims to mimic the intuitive way a cytogeneticist would perform this task, i.e., starting with the cancer karyotype and changing it by “going backwards” towards the normal karyotype one event at a time, taking the simplest and most evident step whenever possible. Often the set of events is uniquely determined, but the order of these events cannot be known from the data. The algorithm terminates successfully if the normal karyotype is reached, and reports “failure” otherwise.

A chromosome is *indefinite* if its description includes unknown items. For example, ?→? and 1pter→1p? are indefinite chromosomes. Note that a definite chromosome may contain uncertain items, e.g. 1pter→1p? 12. Similarly, a karyotype is *definite* if it contains only definite chromosomes. In what follows we analyze only definite karyotypes, and ignore any uncertainties, e.g. 1p? 12 will be considered as 1p12.

An abstract data structure of a karyotype

We represent a karyotype *k* by the following abstract data structure:

- *Abnormal_Chrs(k)*: A set of distinct, orientation-less, abnormal chromosomes. For each abnormal chromosome in *Abnormal_Chrs(k)* we maintain its multiplicity and list of fragments.
- *Normal_Chrs(k)*: a mapping assigning to each normal chromosome *c* (i.e. c=1,...,22,X,Y) its multiplicity in *k*.

Orphan fragments

Denote by *Frags(k)* the multiset of fragments found in *Abnormal_Chrs(k)*. A fragment in *Frags(k)* is orphan if there is no other fragment in *Frags(k)* from the same normal chromosome. For example, suppose *Abnormal_Chrs(k)* = {9pter→9q32::1p36→1pter, 14qter→14p21::9q32→9qter, 14p21→14qter} then *Frags(k)* = {9pter→9q32, 9q32→9qter, 14qter→14p21×2; 1p36→1pter} and *k* contains exactly one orphan fragment: 1p36→1pter.

The algorithm

The SKS algorithm computes a sequence of events S = ρ_1_,...,ρ_t_ that transforms a normal karyotype into a given (cancerous) karyotype *k*. Starting from *k* and applying the corresponding inverse operations S^-1^ = ρ_t_,^-1^...,ρ_1_^-1^ generates a normal karyotype. The events are computed in inverse order, i.e. ρ_t_ is the first computed event.

The SKS algorithm works in two phases. First, all the abnormal chromosomes are sorted. Then, simple numerical operations “correct” the multiplicities of the normal chromosomes. Before describing the algorithm, we need a few definitions. A fragment is *centric* if it contains a centromere, and *acentric* otherwise. Let *f* and *g* be two fragments from the same normal chromosome. The concatenation *f*::*g* is an *adjacency* if *f* and *g* have exactly one shared band - which corresponds to their fused ends. For example, 1pter→1p11::1p11→1q22 is an adjacency. In this case, *f* and *g* are said to be *complementing*. Fragments *f* and *g* in *Frags(k)* are *uniquely complementing* if no other fragment *h* in *Frags(k)* is complementing to *f* or *g*.

**Initialization: Detecting the most recent ploidy change event (if exists).** We first detect a simple global change in the karyotype ploidy as follows. (Additional, more complex ploidy changes are considered at the end of the procedure, see below).Let *m* and *g* be the median and greatest common divisor of all distinct chromosome multiplicities (both normal and abnormal) respectively. Clearly, *m ≥ g*. Suppose *g > 1*. In this case we divide all chromosome multiplicities by *d = g*.

A single exception is when *m= g* and *g* is even - in this case we divide by *d = g/2* (instead of by *g*). If the chromosome multiplicities were changed (i.e. *d > 1*) - we set *S = {ρ}*, where *ρ* is a corresponding PLOIDY_CHANGE event.

**Phase I: Sorting the abnormal chromosome**s. The abnormal chromosomes are sorted by repeatedly detecting and undoing one of the events in Table S1. The phase ends successfully if there are no more abnormal chromosomes, and ends with failure if there are still abnormal chromosomes but no additional event is detected. The events are detected in the priority of the order below. Upon detecting an event ρ that alters an (abnormal) chromosome *c* with multiplicity *n_c_* > 1, the algorithm adds *n_c_-1* CHR_GAIN events of chromosome *c* before ρ.

- INVERSION: An inversion is the reversal of a DNA segment within a chromosome. This event is detected for a pair of uniquely complementing fragments, *f1* and *f2*, on the same chromosome, that have different orientation. The inverse operation is an inversion that fuses the complementing ends of *f1* and *f2*. For example, suppose the chromosome containing fragments *f1* and *f2* is of the form *f1*::*g*::*f2’*::*h*, where *f2’* is the inverse of *f2* and *f1*::*f2* is an adjacency. In this case, the detected INVERSION event inverts the segment *g*::*f2’* resulting in *f1*::*f2*::*g’*::*h*, where *g’* is the inverse of *g*..
- TRASNLOCATION: A translocation is the exchange of tails between two chromosomes. This event is detected for a pair of uniquely complementing fragments, *f1* and *f2*, that are found on two different chromosomes. The inverse operation is a translocation that fuses the complementing ends of *f1* and *f2*. An additional requirement is that undoing the translocation (i.e. applying the inverse translocation) will not result in a new acentric chromosome.
- INSERTION: An insertion is a cut-and-paste of an acentric DNA segment from one chromosome to another. An insertion is identified when all involved ends, i.e. the ends of the inserted fragment and the resulting gap, are uniquely complementing. The inverse operation moves the fragment into its native chromosome.
- TAIL_DEL: A deletion of a chromosome tail (acentric end fragment) is detected by identifying an abnormal chromosome end lacking a pter or a qter, and whose missing tail fragment, *f*, is *(i)* acentric and *(ii)* does not contain in its span any fragment in *Frags(k)*. To undo the operation, concatenate f to the chromosome's end such that a new adjacency is formed.
- IN_DEL: An internal deletion of a fragment within a chromosome is discovered as follows. Detect a non-adjacency pair of concatenated fragments, *f::g*, for which there exists in the normal karyotype an acentric fragment *h* such that *(i)* *f::h* and *h::g* are adjacencies, and *(ii)* *h* does not contain in its span any fragment in *Frags(k)*. Replace *f::g* by fragment *f’≡ f::h::g*.
- TANDEM_DUP: A tandem duplication results in two identical consecutive fragments on the same chromosome, creating *h ≡ f1::f2::f2::f3*. For example, 1pter→1q44::1q31→1qter is a tandem duplication since 1pter→1q44 ≡ 1pter→1q31::1q31→1q44 and 1q31→1qter ≡ 1q31→1q44::1q44→1qter. When identifying such a repetition, simply remove it, forming *h ≡ f1::f2::f3*.
- ISOCHR: Detect any iso-chromosome or iso-derivative, i.e. a chromosome with two identical arms. Perform the inverse operation, by removing one of the identical arms.
- DICENTRIC: Detect a multicentric chromosome *chr* containing a centric orphan *f*. To undo the operation, perform a fission of *chr* near *f* such that each of the resulting two chromosomes contains a centromere.
- TAIL_DUP or CHR_LOSS of a translocation derivative: Detect an acentric orphan fragment, *f*, found on one end of an abnormal chromosome. Suppose *f* is adjacent to fragment *g*.
- If the multiplicity of the normal chromosome of *f* is smaller than the ploidy of *k* – detect a CHR_LOSS of a “complementing” chromosome: *f’::g’* , where *g*’ and *f’* are tail fragments (i.e. containing a pter/ qter end) complementing to *g* and *f* in the normal karyotype respectively.
- Otherwise, detect a TAIL_DUP event and eliminate this aberration by a removal of f.

**Phase II**: **Detection of gain/ loss and ploidy change events**. If this phase is reached then the current karyotype *k* satisfies *Abnormal_Chrs(k) = ∅*. Define *m(k)* as the median multiplicity of all chromosomes in *k* (for gain/ loss computations we consider the sex chromosomes as homologs). For any chromosome *chr* whose multiplicity differs from *m(k)*, adjust its ploidy to *m(k)* by CHR_LOSS or CHR_GAIN events. Then, if the ploidy of all chromosomes is *m(k)*≠2, adjust the ploidy globally to 2 by prepending a corresponding PLOIDY_C HANGE event to S.

[1] Ozery-Flato M, Shamir R.. On the frequency of genome rearrangement events in cancer karyotypes. [Presented in RECOMB satellite conference on Computational Cancer Biology, 2007.](http://casb.ucsd.edu/recombccb07/index.php)  Technical report, Tel Aviv University, http://acgt.cs.tau.ac.il/papers/cancerGR_11b_report-1.pdf (2007)
